# Supplementary material for: Gallbladder adenocarcinomas undergo subclonal diversification and selection from precancerous lesions to metastatic tumors
Source: eLife. 2022 Dec 8;11:e78636. doi: 10.7554/eLife.78636 (PMC9771369; doi:10.7554/eLife.78636)
Supplement: Supplementary file 1. — (A) Baseline characteristics of samples are summarized. (B) Mutational signatures of our dataset are analyzed by three different tools, Mutalisk, Signal, and MuSiCa. [file elife-78636-supp1.docx]

**Supplementary File 1A. Baseline characteristics of samples.**

| **Patient ID** | **Sample** | **Methods for sample acquisition** | **Time of tissue acquisition since initial surgery (months)** | **Tumor purity** | **Type of sample** |
| --- | --- | --- | --- | --- | --- |
| GB-A1 | Normal | Autopsy | 32 | - | Fresh-frozen |
|  | BilIN | Surgery | 0 | 0.30 | FFPE |
|  | GB | Surgery | 0 | 0.35 | FFPE |
|  | Abdominal wall 1 (old) | Surgery | 8 | 0.30 | FFPE |
|  | CBD | Surgery | 9 | 0.40 | FFPE |
|  | Liver 1 | Autopsy | 32 | 0.45 | Fresh-frozen |
|  | Liver 2 | Autopsy | 32 | 0.40 | Fresh-frozen |
|  | Liver 3 | Autopsy | 32 | 0.65 | Fresh-frozen |
|  | Abdominal wall 2 | Autopsy | 32 | 0.30 | Fresh-frozen |
|  | Abdominal wall 3 | Autopsy | 32 | 0.30 | Fresh-frozen |
|  | Abdominal wall 4 | Autopsy | 32 | 0.35 | Fresh-frozen |
|  | Mesentery | Autopsy | 32 | 0.30 | Fresh-frozen |
|  | Omentum 1 | Autopsy | 32 | 0.80 | Fresh-frozen |
|  | Omentum 2 | Autopsy | 32 | 0.25 | Fresh-frozen |
| GB-S1 | Normal | Surgery | 0 | - | FFPE |
|  | BilIN | Surgery | 0 | 0.30 | FFPE |
|  | GB | Surgery | 0 | 0.67 | FFPE |
|  | Regional LN | Surgery | 0 | 0.35 | FFPE |
|  | Distant LN | Surgery | 29 | 0.35 | FFPE |
| GB-S2 | Normal | Surgery | 0 | - | FFPE |
|  | BilIN | Surgery | 0 | 0.25 | FFPE |
|  | GB | Surgery | 0 | 0.50 | FFPE |
|  | Regional LN | Surgery | 0 | 0.25 | FFPE |
| GB-S3 | Normal | Surgery | 0 | - | FFPE |
|  | BilIN | Surgery | 0 | 0.25 | FFPE |
|  | GB | Surgery | 0 | 0.50 | FFPE |
|  | Regional LN | Surgery | 0 | 0.25 | FFPE |
| GB-A2 | Normal | Autopsy | 20 | - | Fresh-frozen |
|  | GB | Autopsy | 20 | 0.66 | Fresh-frozen |
|  | Lung (old) | Surgery | 0 | 0.49 | FFPE |
|  | Lung | Autopsy | 20 | 0.50 | Fresh-frozen |
|  | Chest wall | Autopsy | 20 | 0.43 | Fresh-frozen |
|  | Liver | Autopsy | 20 | 0.67 | Fresh-frozen |
|  | Mesentery | Autopsy | 20 | 0.49 | Fresh-frozen |
| GB-S4 | Normal | Surgery | 0 | - | FFPE |
|  | GB | Surgery | 0 | 0.30 | FFPE |
|  | Colon wall | Surgery | 26 | 0.50 | FFPE |
|  | Distant LN | Surgery | 26 | 0.40 | FFPE |
| GB-S5 | Normal | Surgery | 0 | - | FFPE |
|  | GB | Surgery | 0 | 0.70 | FFPE |
|  | Regional LN | Surgery | 0 | 0.35 | FFPE |
|  | Liver | Biopsy | 11 | 0.48 | FFPE |
| GB-S6 | Normal | Surgery | 0 | - | FFPE |
|  | GB | Surgery | 0 | 0.82 | FFPE |
|  | Regional LN | Surgery | 0 | 0.33 | FFPE |
|  | Lung | Surgery | 0 | 0.76 | FFPE |
| GB-S7 | Normal | Surgery | 0 | - | FFPE |
|  | BilIN | Surgery | 0 | 0.20 | FFPE |
|  | GB | Surgery | 0 | 0.35 | FFPE |
|  | Regional LN | Surgery | 0 | 0.55 | FFPE |
| GB-S8 | Normal | Surgery | 0 | - | FFPE |
|  | BilIN | Surgery | 0 | 0.03 | FFPE |
|  | GB | Surgery | 0 | 0.50 | FFPE |
|  | Regional LN | Surgery | 0 | 0.50 | FFPE |
| GB-S9 | Normal | Surgery | 0 | - | FFPE |
|  | GB | Surgery | 0 | 0.70 | FFPE |
|  | Regional LN | Surgery | 0 | 0.80 | FFPE |
|  | Omentum | Surgery | 0 | 0.85 | FFPE |

BilIN, biliary intraepithelial neoplasia, GB, gallbladder; LN, lymph node; CBD, common bile duct; FFPE, formalin-fixed paraffin-embedded.

**Supplementary File 1B. Mutational signatures of our dataset analyzed by three different tools, Mutalisk, Signal, and MuSiCa.**

|  | **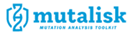** | **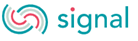** | **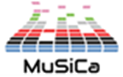** |
| --- | --- | --- | --- |
| **Signature 1** | **43.3%** | **35.7%** | **37.3%** |
| **Signature 3** | **16.4%** | **13.7%** | **7.8%** |
| **Signature 24** | **14.6%** | **8.8%** | **10.7%** |
| **Signature 7** | **13%** | **10.2%** | **10.8%** |
| **Signature 13** | **7.5%** | **6.5%** | **7.1%** |
| **Signature 22** | **5.2%** | **3.3%** | **5.3%** |
| Signature 6 | - | 3.6% | 6.5% |
| Signature 4 | - | 8.8% | - |
| Signature 10 | - | - | 3.5% |
| Signature 17 | - | 3.3% | 3.4% |
| Signature 2 | - | 3.1% | 3.3% |
